# HSPB1 Is Essential for Inducing Resistance to Proteotoxic Stress in Beta-Cells

**DOI:** 10.3390/cells10092178

**Published:** 2021-08-24

**Authors:** Vinícius M. Gomes, Rosangela A. M. Wailemann, Gabriel S. Arini, Talita C. Oliveira, Daria R. Q. Almeida, Ancély F. dos Santos, Letícia F. Terra, Stephan Lortz, Leticia Labriola

**Affiliations:** 1 Biochemistry Department, Chemistry Institute, University of São Paulo, São Paulo 05508000, Brazil; vinickjp@gmail.com (V.M.G.); rosangelawailemann@gmail.com (R.A.M.W.); gabrielbio19@gmail.com (G.S.A.); talita.c.oliveira@gmail.com (T.C.O.); queirozd@ualberta.ca (D.R.Q.A.); ancelybqi@gmail.com (A.F.d.S.); leterra@gmail.com (L.F.T.); 2 Institute of Clinical Biochemistry, Hannover Medical School (MHH), Carl-Neuberg-Straße, 1, 30625 Hannover, Germany

**Keywords:** heat-shock proteins, diabetes mellitus, beta-cells, endoplasmic reticulum stress, proteostasis, HSPB1, cytoprotection, apoptosis

## Abstract

During type 1 diabetes mellitus (T1DM) development, beta-cells undergo intense endoplasmic reticulum (ER) stress that could result in apoptosis through the failure of adaptation to the unfolded protein response (UPR). Islet transplantation is considered an attractive alternative among beta-cell replacement therapies for T1DM. To avoid the loss of beta-cells that will jeopardize the transplant’s outcome, several strategies are being studied. We have previously shown that prolactin induces protection against proinflammatory cytokines and redox imbalance-induced beta-cell death by increasing heat-shock protein B1 (HSPB1) levels. Since the role of HSPB1 in beta cells has not been deeply studied, we investigated the mechanisms involved in unbalanced protein homeostasis caused by intense ER stress and overload of the proteasomal protein degradation pathway. We tested whether HSPB1-mediated cytoprotective effects involved UPR modulation and improvement of protein degradation via the ubiquitin-proteasome system. We demonstrated that increased levels of HSPB1 attenuated levels of pro-apoptotic proteins such as CHOP and BIM, as well as increased protein ubiquitination and the speed of proteasomal protein degradation. Our data showed that HSPB1 induced resistance to proteotoxic stress and, thus, enhanced cell survival via an increase in beta-cell proteolytic capacity. These results could contribute to generate strategies aimed at the optimization of beta-cell replacement therapies.

## 1. Introduction

Pancreatic islet transplantation is an attractive alternative for the treatment of T1DM, recommended mainly for patients suffering from frequent episodes of severe unnoticed hypoglycemia [1,2,3]. Even when several modifications have been adopted to maintain longer survival and functionality of the graft [3,4], the loss of beta-cell mass by autoimmunity, rejection, and instant blood-mediated inflammatory reaction (IBMIR) [5] is still an important factor deserving more attention to increase the success of pancreatic islet transplantation [3,4].

Beneficial effects of prolactin (PRL) or placental lactogen in the process of isolation and culture of these structures during the period prior to transplantation have recently being reported [6,7,8]. Results from our group have shown that PRL was able to induce the upregulation of HSPB1, a protein from the family of small heat-shock proteins (sHSPs), which turned out to be a key mediator of PRL-mediated inhibition of beta-cell apoptosis [9,10]. Heat-shock proteins (HSPs) constitute a large family of highly homologous chaperone proteins that are induced in response to several environmental changes such as elevation of temperature and physical or chemical stresses [11]. In particular, sHSP constitute a very heterogeneous family of ATP-independent chaperones, some of which have been proven to play crucial roles in a wide range of cell types to maintain the integrity and function of tissues, especially that of nervous and muscular tissue [12,13].

It is important to note that we have recently shown that PRL was not able to modulate HSP90 or 70 protein levels in mouse beta-cells [14].

Understanding the mechanisms of beta-cell protection by HSPB1 has crucial importance for future cell-based strategies, since the PRL concentration used to promote beta-cell cytoprotection is not compatible with a clinical application because of the side effects displayed by the hormone in other tissues. It is well known that proinflammatory cytokines such as interleukin 1 β (IL-1-β), interferon-γ (IFN-γ), and tumor necrosis factor-α (TNF-α) can promote beta-cell death by inducing oxidative and endoplasmic reticulum (ER) stress, as well as by promoting a reduced and inappropriate capacity to completely mount a functional autophagic response for protein degradation [15,16]. Our results have demonstrated that increased levels of HSPB1 can inhibit beta-cell apoptosis induced by proinflammatory cytokines, menadione-induced oxidative stress, and thapsigargin-induced ER stress [9,10].

ER stress has been implicated in the pathogenesis of both type 1 and 2 diabetes [17,18,19,20]. Incorrectly folded proteins are retained in the ER to undergo refolding or degradation. Dysregulation of these processes causes ER stress-dependent activation of the unfolded protein response (UPR), which is involved in minimizing stress and restoring homeostasis. Upon prolonged or stronger stress situations UPR can also lead to apoptosis [21,22]. One of the fates of unfolded proteins in the ER is the endoplasmic reticulum-associated protein degradation (ERAD), which promotes the degradation of ubiquitinated proteins by the proteasome system [23]. UPR activation impacts the ERAD pathway, leading to ER stress attenuation and, thus, cell survival [24]. Upon intense induction, and if all the intracellular mechanisms are not able to sustain proper protein quality control, ER stress can finally trigger cell death by inducing apoptosis through the upregulation of CCAAT-enhancer-binding protein homologous protein (CHOP) that counteracts the effect of antiapoptotic proteins. There is substantial evidence for the presence of ER stress in beta-cells, suggesting defective UPR and failure to resolve ER stress in the context of T1DM and T2DM [16,25]. Indeed, CHOP has been proposed as the main link between ER stress induction and beta-cell apoptosis in T1DM [20,26]. Under stress conditions, the induction of CHOP and further activation of BH3-only proteins such as Bcl-2-interacting mediator of cell death (BIM) can cause mitochondrial outer membrane permeabilization and cell death [27,28]. The participation of BIM as a cell death-inducing factor involved in pancreatic beta-cell death has also been reported [16].

In view of the scarce number of studies properly addressing the role of HPSB1 in beta-cells, we set out to unveil the molecular mechanisms involved in HSPB1-induced resistance to proteotoxic stress in insulin-producing cells.

## 2. Materials and Methods

### 2.1. Mouse Islets Isolation and Culture

Islet isolation from male BALB/c mice (8–12 weeks old) was performed according to Wailemann and coauthors [9]. Before terminal surgery, healthy animals (five per cage) were kept in a normal dark/light cycle with ad libitum access to water and food in an environmental enriched ambient in the animal facility of the Chemistry Institute (University of São Paulo, São Paulo, Brazil). Briefly, animals were submitted to terminal surgery for pancreas retrieval after euthanasia by intraperitoneal administration of a lethal dose of anesthetics (xylazine hydrochloride (Kensol, Koenig, Brazil; 10 mg/kg) and ketamine hydrochloride (Vetanarcol, Koenig, Brazil; 100 mg/kg)). The Internal Animal Care and Use Committee of the Chemistry Institute at the University of São Paulo approved all protocols on 23 August 2017 (process n°74/2017) following all norms established by the National Council for the Control of Animal Experimentation. Islets were handpicked and kept in RPMI medium supplemented with 10% (*v*/*v*) FCS, 2 mmol/L glutamine, 100 units/mL ampicillin, and 100 µg/mL streptomycin at 37 °C in a 5% (*v*/*v*) CO_2_ atmosphere.

### 2.2. Cell Culture of Min6 Cells

Mouse insulinoma-derived Min6 cells [29] and all HSPB1-modified cells [10,15] and their respective controls were maintained in RPMI medium (Thermo Fishcer Scientific, Waltham, MA, USA) supplemented with 10% (*v*/*v*) FCS, 100 units/mL ampicillin, 100 µg/mL streptomycin, and 10 mmol/L HEPES (Sigma-Aldrich, St. Louis, MO, USA). Cell lines were periodically submitted to PCR and Hoechst staining to analyze mycoplasma contamination and were only used in case of negative results.

### 2.3. HSPB1 Silencing and Overexpression in Mouse Islets

#### 2.3.1. HSPB1 Silencing

Dispersed islets were infected with (MOI: 4.6) recombinant lentiviral particles containing a mixture of five validated shRNAs for murine hspb1 (Santa Cruz Biotechnology, Santa Cruz, CA, USA) or the corresponding controls as previously described [30,31].

#### 2.3.2. Transient HSPB1 Overexpression

pEGFPhsp27 wt FL, a gift from Andrea Doseff (#17444; Addgene, Cambridge, MA, USA) [32], or the empty vector pEGFP (#19056; Addgene, Cambridge, MA, USA) was used to transfect mouse islets as previously described [9]. Transfection was performed using Lipofectamine RNAiMAX (Thermo Fisher Scientific). Lipid-DNA complexes (0.2 μL of Lipofectamine: 500 ng of plasmid) were formed in Opti-MEM (Thermo Fisher Scientific) at room temperature for 20 min and added to primary cultures of mouse islets in antibiotic-free medium for overnight transfection. Islet cells were maintained in culture for a 24 h recovery period before experiments were carried out. Transfection efficiency was validated by Western blot (WB) and by monitoring eGFP fluorescence by epifluorescence microscopy as previously described [9].

### 2.4. Cell Treatments

Cells were seeded at different concentrations depending on further experimentation and allowed to attach for a period of 24 h. Cells were then serum-starved for 24 h in the respective medium supplemented with 0.1% (*v*/*v*) FCS. Treatments with a cocktail of proinflammatory cytokines (IL-1β, 1.6 ng/mL; TNF-α, 8 ng/mL; IFN-γ, 16 ng/mL), tunicamycin (mouse islets:7.5 or Min6 cells: 15 µg/mL), or thapsigargin (75 nM) (both from Sigma-Aldrich) were carried on for 16 h (for cell viability) or 30 min, 1 h, 3 h, 6 h, and 9 h (for protein extraction). The proteasome activity was inhibited by preincubating the cells for 1 h with MG132 (0.1 or 1 μM, Calbiochem, La Jolla, CA, USA). This inhibitor was maintained during the subsequent incubations with the cytokine cocktail, ER stress inducers, or PRL. PRL (300 ng/mL; Peprotech, Mexico) incubations were started 30 min before the addition of either cytokines or ER stress inducers and kept through the whole treatment.

### 2.5. Cell Viability Evaluated by Microscopy

Cells were stained with 15 µg/mL propidium iodide (PI, Sigma Aldrich) and 15 µg/mL Hoechst 33,342 (HO, Sigma-Aldrich) for 10 min. The percentage of viable and dead cells was determined as already described [9]. A minimum of 500 cells was counted for each experimental condition.

### 2.6. Glucose-Induced Insulin Release Assay

An equal number of primary mouse islets were preincubated for 1 h in the presence or in the absence of recombinant human prolactin (PRL). After this period, cells were exposed to the cytokine cocktail for 16 h in RPMI medium supplemented with 0.1% FCS with or without PRL. Cells were then incubated with Krebs–Ringer buffer supplemented with 0.2% bovine serum albumin (BSA) and 5.6 mM glucose for 30 min at 37 °C. The buffer was replaced with Krebs buffer 0.2% BSA supplemented with 2.8 mM glucose for 1 h. The supernatant was collected, and the cells were placed in Krebs–Ringer buffer (0.2% BSA supplemented with 16.7 mM glucose) for 1 h. This solution was collected, and the cells were subjected to an acidic ethanol solution (HCl 1.4%, ethanol 74.3%, Milli-Q water 24.3%) to determine total insulin content. Total DNA content was further extracted by citrate buffer (citric acid 3.15 g/L, NaCl 8.77 g/L, disodium EDTA 1.008 g/L, pH 7.4). All chemicals used were of analytical grade and were purchased from Sigma Aldrich, unless otherwise stated. The collected solutions were stored at −20 °C until insulin was quantified by ELISA (Mercodia, Stockholm, Sweden). The values were normalized by DNA content using spectrofluorimetry (SpectraMax M2, Molecular Devices, San Jose, CA, USA) as previously described [33].

### 2.7. Western Blots

Total cell lysates were prepared from cells plated at a density of 3 × 10^4^ cells/cm^2^.

Equal amounts of proteins from each extract were solubilized in sample buffer (60 mmol/L Tris-HCl (pH 6.8), 2% SDS, 10% glycerol, 0.01% bromophenol blue) and subjected to SDS-PAGE (10–16%). Proteins were transferred to PVDF membranes, which were blocked and then incubated with the antibodies listed in Supplementary Table S1. Detection was performed by enhanced chemiluminescence (Millipore, Billerica, MA, USA) using horseradish peroxidase-conjugated secondary antibodies (Vector Laboratories, Burlingame, CA, USA). As a loading control, the membranes were stripped and re-probed with mouse monoclonal anti-α-Tubulin (B512) (Sigma-Aldrich). For the analysis of protein phosphorylation levels, the membranes were stripped and re-probed with the corresponding anti-total protein. Quantitative densitometry was carried out using ImageJ software (National Institutes of Health, Bethesda, MD, USA). The volume density of the chemiluminescent bands was calculated as an integrated optical density × mm^2^ after background correction from at least two different images from each independent experiment (*n* ≥ 3).

### 2.8. Study of the Rate of Protein Degradation by the Ubiquitin-Proteasome System

The rate of protein degradation by the ubiquitin-proteasome system was measured by checking the levels of the GFP protein whose sequence was modified (G76V) so that it can be easily ubiquitinated and consequently degraded by the proteasome system, according to the methodology described by Dantuma and coworkers [34,35]. Briefly, lipid–DNA complexes were formed in Opti-MEM in a proportion of 0.2 µL of Lipofectamine for 500 ng of plasmid at room temperature for 20 min, and then added to the cells in antibiotic-free medium for 16 h. The medium was then replaced by RPMI containing 10% FCS. Min6 oxHSPB1 and EV cells were subjected to the UbG76V-GFP (GFP-Ub) vector transfection process performed using Lipofectamine RNAiMAX. Cells were serum-starved and incubated for 3, 6, and 9 h with proinflammatory cytokines, ER stressors, or vehicle. Total protein extracts were obtained, and GFP and α-tubulin levels were detected by Western blot. Since the speed of GFP degradation reflects the ubiquitin-proteasome protein degradation pathway activity [34,35], the rate of GFP degradation was measured by calculating the area under the normalized curve, which shows the amount of protein normalized to the α-tubulin content present throughout the treatment time.

### 2.9. Statistical Analysis

Statistical analyses were performed using GraphPad Prism version 6.0 software (Graphpad Sofware, San Diego, CA, USA). All results were analyzed for Gaussian distribution and passed the normality test. In cell viability tests, the statistical differences between the means of the experimental groups were tested through one-way ANOVA analysis followed by the Tukey post hoc test for multiple comparisons. The statistical differences between protein levels of the different cell lines submitted to treatments at different times were tested by two-way ANOVA analysis followed by Bonferroni’s post hoc test for multiple comparisons. For all tests, a value of *p* < 0.05 was considered to be statistically significant.

## 3. Results

### 3.1. HSPB1 Is Required for Beta-Cell Cytoprotection against ER Stress-Induced Apoptosis

**Figure 1:**
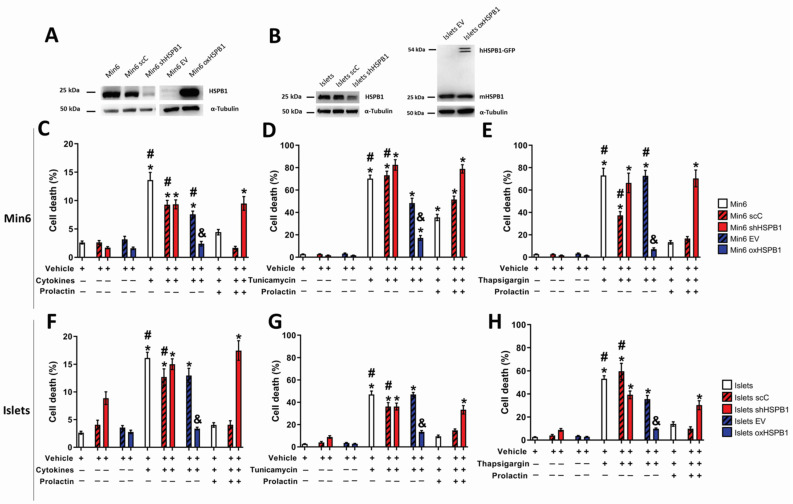
HSPB1 is a key mediator of prolactin-mediated cytoprotection against ER stress in Min6 cells and mouse islets. HSPB1 expression was confirmed by Western blot in (**A**) Min6 cells and (**B**) mouse islets. HSPB1-silenced (Min6 shHSPB1, Islets shHSPB1) and HSPB1-overexpressing (Min6 oxHSPB1, Islets oxHSPB1) Min6 cells and mouse islets, as well as their respective controls (Min6 scC, Min6 EV; Islets scC, Islets EV), were exposed to a combination of cytokines (**C**,**F**) (TNF-α 8 ng/mL, INF-γ 16 ng/mL, IL-1-β 1.6 ng/mL), (**D**,**G**) tunicamycin (15 or 7.5 µg/mL, respectively), or (**E**,**H**) thapsigargin (75 nM) in the presence or absence of PRL (300 ng/mL) for 16 h. Cell death was evaluated by PI/Hoechst staining using fluorescent microscopy. Results are presented as means ± SEM (each data point represents the mean ± SEM from three replicates of at least three independent experiments); * *p* < 0.05 vs. control (vehicle); ^#^ *p* < 0.05 vs. PRL; ^&^ *p* < 0.05 vs. EV.

Since we have shown that HSPB1 was involved in prolactin-promoted cytoprotection in the context of T1DM and ER stress-induced cell death by thapsigargin [9], we investigated the role of this chaperone in ER stress-induced cell death. Min6 cells and primary cultures of mouse islets were challenged with a combination of proinflammatory cytokines that are responsible for inducing ER stress in beta-cells during T1DM [17] or endoplasmic reticulum stressors: tunicamycin or thapsigargin for 16 h in the presence or absence of PRL. Results displayed in Figure 1A,C–E showed that prolactin was able to inhibit apoptosis by approximately 50% in all cell lines expressing HSPB1 (Min6, Min6 scC, and Min6 EV). This effect was abrogated in the absence of the chaperone (Min6 shHSPB1). Additionally, overexpression of HSPB1 (Min6 oxHSPB1) was enough to protect beta-cells against induced death independently of hormonal treatment. A more pronounced HSPB1-dependent cytoprotective effect was observed in primary cultures of mouse islets overexpressing the chaperone (Islets oxHSPB1) (Figure 1B,F–H).

Of note is the fact that modulation of HSPB1 levels did not promote significant changes in glucose-induced insulin secretion or insulin content in Min6 cells [14] or in primary cultures of mouse islets (Supplementary Figure S1). In sum, we showed that HSPB1 silencing or overexpression did not hamper the secretory function of beta-cells.

These results demonstrated that HSPB1 is an essential player in the PRL-induced beta-cell cytoprotection against ER stressors’ cytotoxic properties. Moreover, the sole overexpression of this protein was sufficient to provide a significant prosurvival effect independent of PRL treatment.

### 3.2. HSPB1 Modulates UPR in Mouse Islets and Min6 Cells

To investigate the molecular mechanisms leading to the cytoprotective effect of HSPB1, protein levels of several components of the UPR pathways were analyzed.

Since we have already shown that caspase-3 activity reached a plateau upon 9 h of cytokine treatment [9], protein levels and phosphorylation states were analyzed in kinetic experiments (0–9 h). The quantitative analyses between the cells were performed by comparing protein expression levels 6 or 9 h after ER stress stimuli.

**Figure 2:**
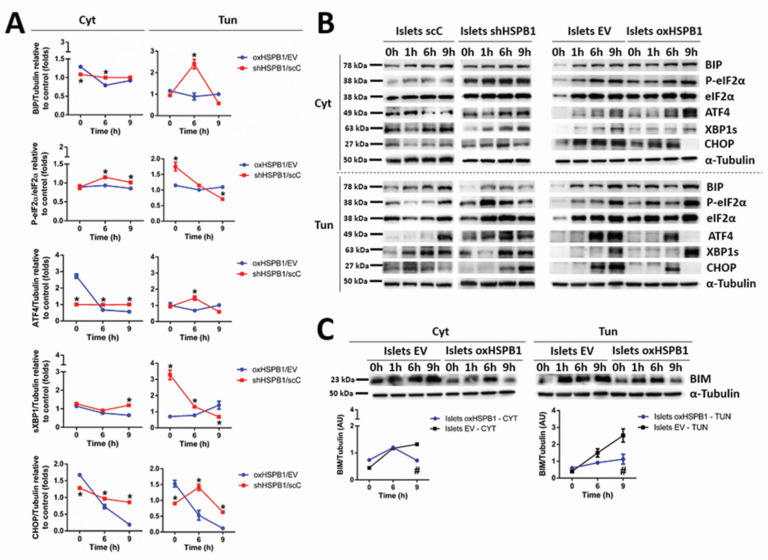
HSPB1 overexpression attenuates ER stress induced by proinflammatory cytokines or tunicamycin in mouse islets. HSPB1-silenced (shHSPB1) and HSPB1-overexpressing (oxHSPB1) mouse islets, as well as their respective controls (scC, EV), were exposed to a combination of cytokines (TNF-α 8 ng/mL, IFN-γ 16 ng/mL, IL-1-β 1.6 ng/mL) or tunicamycin (7.5 µg/mL) for 6 and 9 h. Protein expression of BIP, ATF4, XBP1s, and CHOP, as well as phosphorylation state of eIF2α, was analyzed by Western blotting. (**A**) Graphical representation of BIP, eIF2α, ATF4, XBP1s, and CHOP protein levels presented as arbitrary densitometry units (AU). After normalization to the corresponding α-tubulin content, the data of the silenced (Min6 shHSPB1, Islets shHSPB1) or overexpressed (Min6 oxHSPB1, Islets oxHSPB1) HSPB1 cells were plotted as the ratio between the values obtained in silenced or overexpressing cells and those in their respective controls (scC or EV). (**B**) Immunoblots of islets are shown as representative results. (**C**) Graphical representation of normalized protein levels and immunoblots of BIM protein levels in islets presented as arbitrary densitometric units (AU). Each data point represents the mean ± SEM from three replicates and at least three independent experiments carried out for each cell type submitted to the different cell incubation conditions; * *p* < 0.05 vs. oxHSPB1/EV; ^#^ *p* < 0.05 vs. EV.

HSPB1-overexpressing cells displayed significant stress attenuation. The first evidence of this effect was the lower level of binding immunoglobulin protein (BIP) observed in oxHSPB1 islets, as well as in Min6 oxHSPB1 cells, after 6 h of proinflammatory cytokines or tunicamycin exposure when compared with the silenced cells (islets shHSPB1 and Min6 oxHSPB1, respectively) (Figure 2 and Supplementary Figure S2).

Regarding the Protein kinase RNA-like endoplasmic reticulum kinase–eukaryotic initiation factor 2 alpha–Activating transcription factor 4 (PERK-eIF2α-ATF4) UPR branch, we detected increased PERK phosphorylation levels upon 9 h of islets oxHSPB1 exposure to either of the ER stressors when compared with silenced cells (Figure 2). This effect was also observed in Min6 oxHSPB1 cells treated with proinflammatory cytokines (Supplementary Figure S3). It is important to note that this last model showed an earlier response since the increase was already significant after 6 h of treatment (Supplementary Figure S2). Phosphorylated eIF2α (P-eIF2α) levels were decreased in both cell models overexpressing HSPB1 already after 6 h of cytokine exposure and remained at lower levels until 9 h of treatment (Figure 2, Supplementary Figure S2). Tunicamycin-exposed cells, whether Islets oxHSPB1 or Min6 oxHSPB1 cells, displayed a significant decrease in P-eIF2α only after 9 h of ER stress induction. Lastly, unlike Min6 oxHSPB1, Islets oxHSPB1 also presented reduced levels of ATF4 after 6 h of exposure to any of the ER stressors. This same profile was maintained until the last timepoint analyzed (Figure 2, Supplementary Figure S2). Overall, these results led us to conclude that higher abundance of HSPB1 promoted the attenuation of the UPR pathway initiated by PERK activation in both primary mouse islets and Min6 cells.

Phosphorylation levels of inositol-requiring enzyme 1 alpha (pIRE1α), as well as spliced X-box-binding protein 1 (XBP1s), were differently modulated in HSPB1-overexpressing cells depending on the ER stressor and on the cell model used. While exposure to the cytokine’s cocktail promoted an increase in pIRE1α, tunicamycin treatment led to lower phosphorylation levels of the protein in both cell models bearing higher chaperone levels (Supplementary Figures S2 and S3). Of note are the slightly different kinetics displayed by mouse islets and Min6 cells. While, in primary mouse islets, the effect appeared to be transient, in the latter model, the effect was more sustained (Supplementary Figures S2 and S3). When we studied the abundance of IRE1α’s downstream effector, XBP1s, significantly lower levels were detected in islets oxHSPB1 after 9 h of cytokines exposure. Only a trend of decrease was detected in Min6 oxHSPB1 (Figure 2 and Supplementary Figure S2). Regarding tunicamycin treatment, both cell types presented significantly diminished levels of the spliced form of the protein upon this strong and specific ER stress induction (Supplementary Figures S2 and S3). A delayed response was observed in Min6 oxHSPB1cells when compared with the earlier decrease displayed by primary mouse islets (Supplementary Figures S2 and S3). Since activation of both PERK and IRE1α signaling pathways can lead to upregulation of CHOP [16], which then leads to an increase in BH3 proteins involved in apoptosis activation [36,37], we analyzed the levels of some of these proteins in our HSPB1-overexpressing and -silenced cell models.

An approximately fourfold decrease was observed in proapoptotic CHOP protein levels in mouse islets exposed to proinflammatory cytokines (oxSHPB1/EV: 0.18 ± 0.03 (9 h); shHSPB1/scC: 0.85 ± 0.01 (9 h)) or tunicamycin (oxSHPB1/EV: 0.12 ± 0.03 (9 h); shHSPB1/scC: 0.62 ± 0.01 (9 h)) (Figure 2). A greater reduction in the levels of the same protein was observed in Min6 oxHSPB1cells (Supplementary Figure S2). It is important to highlight that the response promoted by HSPB1 overexpression was completely lost in HSPB1 silenced cells (shHSPB1/scC) (Figure 2A,B, Supplementary Figure S2). Of note are also the significantly lower levels of CHOP’s target BIM (EV: 1.32 ± 0.05 (9 h); oxHSPB1: 0.71 ± 0.03 (9 h)) observed in Islets oxHSPB1 (Figure 2C).

Additionally, decreased levels of cleaved ATF6, another factor promoting CHOP upregulation, were detected in MIN6 oxHSPB1 cells after 9 h of cytokines or tunicamycin exposure (Supplementary Figure S2). All together, these results further confirmed our hypothesis that HSPB1 overexpression in insulin-producing cells is able to attenuate ER stress-induced UPR activation.

In conclusion, despite some discrepancies in the response of the different cell models, these data highlight the importance of increased HSPB1 expression in order to promote ER stress attenuation compatible with the restoration of cell homeostasis. The observed UPR modulation included a reduction in proapoptotic factors such as CHOP and BIM, leading to the interruption of the main link between UPR and apoptosis.

### 3.3. HSPB1-Mediated Beta-Cell Survival Requires a Functional Ubiquitin-Proteasome Protein Degradation System

One of the fates of unfolded proteins in the ER is the ERAD pathway, which is mediated by the proteasome system [38,39].

**Figure 3:**
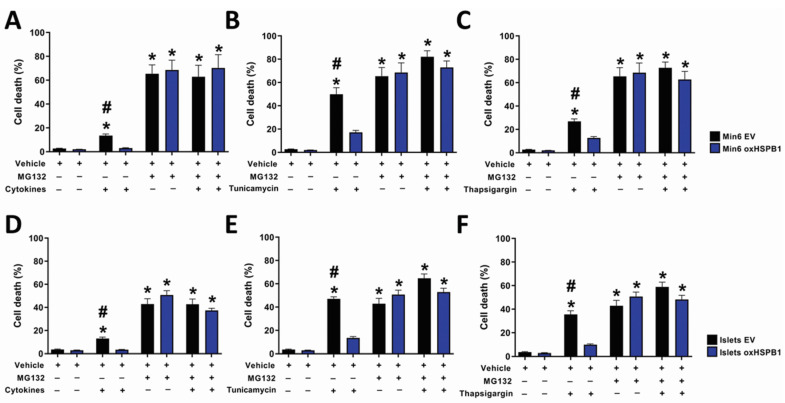
Proteasomal activity is essential for the cytoprotective effect of HSPB1 against cytokine-, tunicamycin- or thapsigargin-induced cell death. (**A**–**C**) HSPB1-overexpressing Min6 cells and (**D**–**F**) mouse islets (Min6 oxHSPB1, Islets oxHSPB1) and their respective controls (Min6 EV, Islets EV) were submitted to serum starvation (0.1% FCS) and then exposed to a combination of (**A**,**D**) cytokines (TNF-α 8 ng/mL, INF-γ 16 ng/mL, IL-1β 1.6 ng/mL), (**B**,**E**) tunicamycin (15 or 7.5 µg/mL respectively), or (**C**,**F**) thapsigargin (75 nM) in the presence or absence of MG132 (2 μM) for 16 h. Cell death was evaluated by PI/Hoechst staining using fluorescent microscopy. Each data point represents the mean ± SEM from three replicates and at least three independent experiments carried out for each cell type submitted to the different cell incubation conditions; * *p* < 0.05 vs. control (vehicle); ^#^ *p* < 0.05 vs. oxHSPB1 respective treatment.

In order to confirm whether this degradation process was an essential mechanism involved in the cytoprotective effect induced by HSPB1 upregulation, the cells were incubated in the presence or absence of the proteasome inhibitor MG132 and then treated with proinflammatory cytokines or ER stressors. Exposure to the proteasome inhibitor alone promoted significant cell death (Supplementary Figure S4). Interestingly, this effect in Min6 shHSPB1 cells was the highest among all the cells tested. Independently of HSPB1 overexpression, cell death rate was further increased when Min6 or islet cells were treated with cytokines or tunicamycin in the presence of the proteasome inhibitor (Figure 3). These results suggest not only that proteasome activity is important to maintain cell viability but also that this system is essential for the cytoprotection mediated by HSPB1 overexpression.

**Figure 4:**
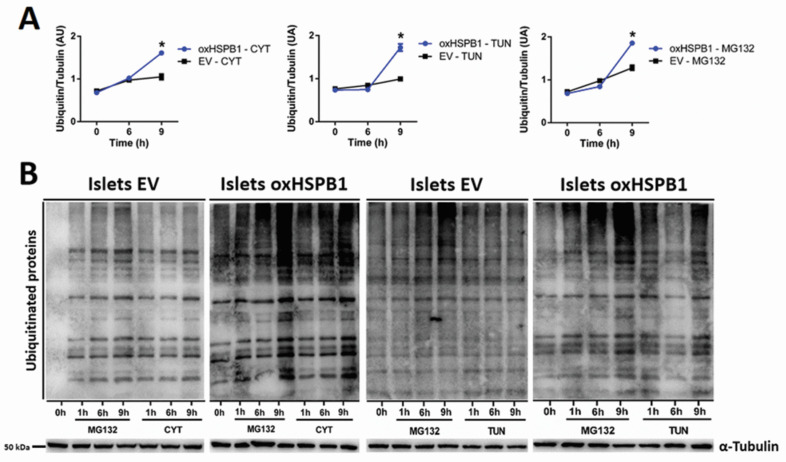
HSPB1 overexpression leads to increased global protein ubiquitination. Mouse islets overexpressing HSPB1 (oxHSPB1) and the respective controls (EV) were serum-starved and then exposed to a combination of cytokines (TNFα 8 ng/mL, IFNγ 16 ng/mL, IL-1β 1.6 ng/mL), tunicamycin (7.5 µg/mL), or MG132 (2μM) for 6 and 9 h. (**A**) Graphical representation of ubiquitinated proteins results presented as arbitrary densitometry units (AU) after normalization by their respective α-tubulin contents. (**B**) Representative images of immunoblots showing the levels of ubiquitinated proteins of islets treated with cytokines and tunicamycin. Each data point represents the mean ± SEM from three replicates and at least three independent experiments carried out for each cell type submitted to the different cell incubation conditions; * *p* < 0.05 vs. EV.

We then assessed whether protein ubiquitination was involved in HSPB1-mediated beta-cell survival. A significant increase in protein ubiquitination was detected in Islets oxHSPB1 incubated with cytokines or tunicamycin after 6 and 9 h (Figure 4). The same effect was displayed by Min6 oxHSPB1cells compared with silenced cells (Supplementary Figure S5).

As a positive control, a significant increase in ubiquitinated proteins was also observed when proteasomal activity was pharmacologically inhibited (Figure 4, Supplementary Figure S4). These data confirmed that HSPB1 overexpression was responsible for the higher protein ubiquitination as part of the cell response involved in HSPB1-mediated beta-cell survival.

### 3.4. HSPB1 Accelerates the Rate of Proteasomal Protein Degradation

**Figure 5:**
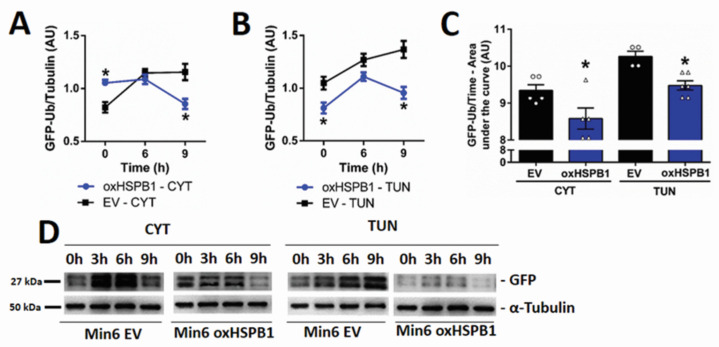
HSPB1 overexpression accelerates the rate of protein degradation by decreasing the levels of ubiquitinated proteins in Min6 cells submitted to ER stress. Min6 OX and Min6 EV cells were transfected with the GFP-Ub vector and were then exposed to a combination of proinflammatory cytokines (TNF-α 8 ng/mL, IFN-γ 16 ng/mL, IL-1β 1.6 ng/mL) or tunicamycin (15 µg/mL). GFP levels were analyzed in total protein extracts obtained after 6 and 9 h of treatment and analyzed by Western blot assays. Quantification of the GFP levels presented as arbitrary densitometric units (AU) after normalization to the corresponding α-tubulin content in cells treated with (**A**) cytokines or (**B**) tunicamycin. (**C**) Histogram representation of the area under the GFP level curves of the different cell types submitted to ER stressors for 9 h. (**D**) Representative images of the immunoblots showing GFP expression in Min6 cells treated with cytokines or tunicamycin. Each data point represents the mean ± SEM from three replicates and at least three independent experiments carried out for each cell type submitted to the different cell incubation conditions; * *p* <0.05 vs. EV.

In view of the above reported results, the following step was to evaluate the impact of this chaperone on the rate of protein degradation via the proteasome system using a reporter strategy. This included the transient transfection of cells with an expression vector containing a modified version of GFP that is rapidly polyubiquitinated and, thus, targeted to degradation [34,35]. By monitoring the levels of this modified GFP over time, we were able to assess changes in protein degradation rates. Min6 oxSHPB1 cells showed significant lower GFP levels than those observed in Min6 EV after 9 h of cytokines (EV: 1.15 ± 0.07; oxHSPB1: 0.85 ± 0.04) or tunicamycin (EV: 1.36 ± 0.08; oxHSPB1: 0.95 ± 0.05) treatment (Figure 5A,B). We also assessed this parameter by analyzing the areas under the curves that integrate GFP levels during the whole period of treatment. We obtained smaller values for Min6 oxHSPB1 compared with Min6 EV cells after ER stress induction either by cytokine (oxHSPB1: 8.58 ± 0.28; EV: 9.34 ± 0.15) or tunicamycin (oxHSPB1: 9.47 ± 0.12; EV: 10.26 ± 0.14) (Figure 5C). These results suggested that HSPB1 overexpression was able to increase the rate of proteasomal protein degradation in cells exposed to ER stressors, further contributing to an adaptive response leading to manageable ER stress and, thus, avoiding the fatal fate observed in wild-type and further enhanced in silenced cells.

## 4. Discussion

In the present study, we were able not only to validate that HSPB1 is a key mediator of the cytoprotective effects mediated by prolactin in type 1 diabetes context [9,10], but also to demonstrate that HSPB1 upregulation, independently of PRL treatment, is sufficient to protect beta-cells and murine islets against ER stress-induced cell death. Moreover, this protein was capable of attenuating the UPR response by preventing the raise of the proapoptotic proteins CHOP and BIM. We have also shown that one of the HSPB1 cytoprotective mechanisms relies on the modulation of the proteasomal degradation pathway by enhancing not only the overall protein ubiquitination but also the degradation rate.

Increased expression of heat-shock proteins is known to mediate the attenuation of various types of stress, including UPR activation and ER stress [40], thus promoting cell survival [41]. It was recently demonstrated that prolactin and lactogen hormones protect INS1 cells, primary islets from Akita mice, and human islets against cell death induced by tunicamycin and thapsigargin, by modulating UPR and attenuating proapoptotic proteins [8]. This effect was completely abrogated by STAT inhibition. These results further reinforce ours since we have previously shown, using human and mouse islets, that already after 2 h of PRL treatment HSPB1 protein expression was increased. This step was essential for cytoprotection and depended on JAK–STAT activation [9,42,43]. In the present study, we further demonstrated that just HSPB1 overexpression is enough to improve cell viability via ER stress attenuation and beta-cell death inhibition.

A reduction of UPR pathways mediating cell death such as PERK/eIF2α/ATF4, IRE1/XBP1s/CHOP, and IRE1α/ASK1/JNK observed in the present study could be pointed out as one of the reasons for increased beta-cell viability promoted by HSPB1 overexpression. It has been reported already that ATF4 regulates the expression of the proapoptotic factor CHOP, which is the main link between UPR and beta-cell apoptosis [44,45]. This, in turn, upregulates BIM, another factor directly linked to beta-cell death [46]. The attenuation of IRE1α phosphorylation may also be linked to a lower activation of the IRE1/XBP1s/CHOP pathway. It is known that, in situations of prolonged neuronal stress, XBP1s can increase the expression of the proapoptotic factor CHOP [44]. A recent report using mouse beta-cells showed that overexpression of XBP1s was associated with increased apoptosis and impairment of insulin expression, as well as glucose-stimulated insulin secretion [47]. Sharma and collaborators have unveiled the crosstalk between ATF6 activation pathway and XBP1 targets in mouse islets submitted to ER stressors [48]. It is important to note that, in their report, ER stress was not induced with proinflammatory cytokines but with thapsigargin or tunicamycin. In their case, despite certain differences in the kinetics of the process, this crosstalk was independent of the ER stressor used [48]. Our results showed that apoptosis inhibition observed in two different models of insulin-producing cells overexpressing HSPB1 could be associated with a decrease in cleaved ATF6 and XBP1s levels starting from 6 h and lasting at least 9 h. This effect was also cell type- and stressor-dependent. It is important to take into account that Min 6 cells are cells derived from an insulinoma [29] and, among the landmarks of cancer cells, a greater resistance to avoid cell death pathways is one of them.

The activation of the IRE1α–ASK1–JNK signaling pathway has also been postulated as a cell death trigger. JNK is a proapoptotic protein kinase that leads to the inhibition of antiapoptotic BCL-2 proteins by phosphorylating them and, thus, inducing cell death in HEK 293T, lung cancer (A549) cells [49,50], hepatocytes [51], and beta-cells [52]. Like HSPB1, exendin-4 was reported to alleviate ER stress induced by exposure of rat insulinoma cells to high glucose and palmitate. This attenuation was mediated by a decrease in PERK, eIF2α, IRE1α, and JNK phosphorylation, as well as reduced ATF6 levels [53].

HSPB1 overexpression promoted, in general, an attenuation of this pathway in murine pancreatic islets. This effect can be associated with a decrease in unfolded proteins in the ER through activation of the ERAD that translocates nonfunctional proteins to the cytosol, which in turn will be ubiquitinated and directed to proteasomal degradation [23]. The synthesis and the folding of insulin place a significant demand on beta-cells, and failure to adapt to ER stress contributes to loss of function and beta-cell death [54,55,56,57]. Indeed, the increase in protein ubiquitination and speed of degradation observed in insulin-producing cells overexpressing HSPB1 in this study further corroborate this hypothesis. Moreover, the interactome data obtained by our research group showed that only when PRL treatment promoted the upregulation of HSPB1 was this chaperone able to interact with enzymes involved in the ubiquitination of proteins, as well as with several catalytic subunits of the proteasome in Min6 cells incubated with proinflammatory cytokines [10]. Thus, one can state that, by decreasing the levels of unfolded proteins in the ER, the activation of the UPR may be significantly attenuated by HSPB1. Indeed, it has been implicated that UPR coupled with ERAD promotes better cell survival by mitigating ER stress [58,59,60]. In this scenario, heat-shock protein 22 (HSP22) has been involved in protein degradation as an adapter protein between the unfolded substrate and the ubiquitination complex in rat cardiac myocytes [61]. There is also evidence that, in cancer cell models, HSPB1 is able to improve proteasome stability, allowing an increase in the complex ability to degrade proteins during ER stress by UPR [62]. Degradation of proapoptotic proteins may also be one of the pathways for improving cell survival. HSPB1 has been reported to participate in the degradation process of the proapoptotic factor BIM, promoting greater survival of pheochromocytoma-derived PC12 tumor cells subjected to ER stress [63]. In the context of T1DM, the upregulation of BIM has already been reported as being part of the signals involved in beta-cell apoptosis induction [64,65].

HSPB1 was shown to increase the degradation of ubiquitinated proteins in response to stress stimuli triggered by TNF-α when interacting with the 26S portion of the proteasome in human leukemia (U937), murine embryogenic fibroblast, and rat colon carcinoma cells [66].

Our previous studies of the HSPB1 interactome in beta-cells have shown that HSPB1 interacts mainly with several catalytic subunits of the proteasome [10]. In the present study, we demonstrated that HSPB1-induced beta-cell survival depends on proteasomal activity. The high sensitivity of beta-cells to ER stress-induced apoptosis can be related to the synthesis and secretion of insulin [67], requiring an efficient cellular protein translation quality control machinery. Disturbances can lead to a rapid activation of cell death pathways [68].

**Figure 6:**
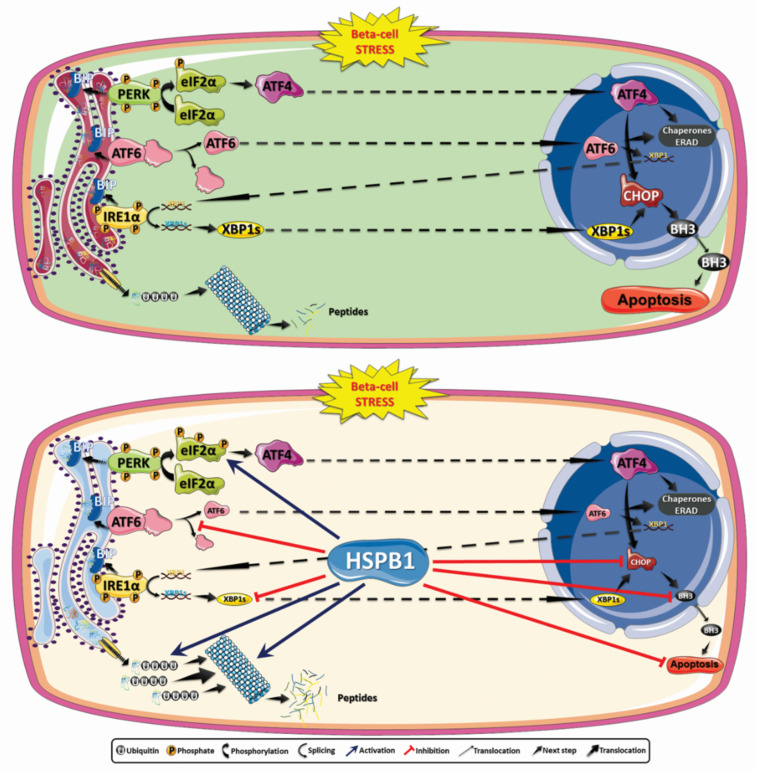
HSPB1 inhibits ER stress-induced cell death by modulating UPR, protein ubiquitination, and protein degradation rate via the proteasome. The UPR is activated by stress to restore cellular homeostasis. (**Upper panel**) Under conditions of intense and prolonged stress, UPR activates mechanisms of cell death by increasing the expression of proapoptotic proteins such as CHOP and proteins of the BH3-only family. (**Bottom panel**) HSPB1 overexpression in mouse pancreatic islets promotes increased PERK phosphorylation, as well as attenuation of eIF2α phosphorylation, ATF4 and cleaved ATF6 levels, the IRE1α pathway, and expression of proapoptotic mediators CHOP and BIM. This process, associated with an increase in protein degradation by the ubiquitin-proteasome system, allows keeping cellular proteostasis under control, thus avoiding the activation of proapoptotic signaling pathways.

Altogether, our data contributed to unveil a cytoprotective molecular mechanism where HSPB1 overexpression promotes UPR modulation, resulting mainly in ER stress attenuation by increasing protein ubiquitination and proteasomal protein degradation. Overall, this process led to decreased levels of the key players linking ER stress and apoptosis such as CHOP and BIM, thus contributing to beta-cell protection (Figure 6).

Collectively, our results provide deeper knowledge of HSPB1 action in beta-cells submitted to ER stress. They underscore the importance of further studies for the development of strategies to mitigate beta-cell death independently of the immune system modulation by upregulating HSPB1-activated endogenous protection pathways, thus paving the way for new therapeutic alternatives improving the outcome of islet transplantation by increasing beta-cell viability.

## Data Availability

All data presented in this manuscript are available upon request to the corresponding author.

## Supplementary Materials

The following data are available online at https://www.mdpi.com/article/10.3390/cells10092178/s1: Supplementary Table S1. List of primary antibodies used for protein detection by Western blot; Supplementary Figure S1. Secretory function and insulin content of primary cultures of mouse islets; Supplementary Figure S2. Increased HSPB1 expression modulates the UPR in Min6 cells under ER stress induced by proinflammatory cytokines or tunicamycin; Supplementary Figure S3. Increased HSPB1 expression modulates PERK and IRE1α phosphorylation in mouse islets under ER stress induced by proinflammatory cytokines or tunicamycin; Supplementary Figure S4. The presence of HSPB1 promotes a longer survival of murine pancreatic beta-cell with inhibited proteasome; Supplementary Figure S5: HSPB1 levels modulate global protein ubiquitination in Min6 cells; Supplementary Figure S6: Uncropped blots.
