# Supplementary material for: HSPB1 Is Essential for Inducing Resistance to Proteotoxic Stress in Beta-Cells
Source: Cells. 2021 Aug 24;10(9):2178. doi: 10.3390/cells10092178 (PMC8472426; doi:10.3390/cells10092178)
Supplement: Supplementary file 1 [file cells-10-02178-s001.zip › cells-1311263-supplementary.pdf]

Supplementary Material

Supplementary Table S1. List of primary antibodies used for protein detection by Western blot

Antibody	Specificity	Order Nr	Company	Dilution
Anti-Phospho-IRE1α	Monoclonal	ab48187	Abcam[1]	1:500
Anti-HSP27 (HSPB1)	Polyclonal	ab5579	Abcam[1]	1:1000
Anti-ATF6	Monoclonal	sc-22799	Santa Cruz[2]	1:1000
Anti-HSP27 (HSPB1)	Monoclonal	sc-13132	Santa Cruz[2]	1:1000
Anti-α-Tubulin	Monoclonal	B512	Sigma-Aldrich[3]	1:100.000
Anti-BIM	Polyclonal	2819	Cell Signaling[4]	1:1000
Anti-BIP	Polyclonal	3183	Cell Signaling[4]	1:1000
Anti-Ubiquitin	Polyclonal	3933	Cell Signaling[4]	1:1000
Anti-ATF4	Monoclonal	11815	Cell Signaling[4]	1:1000
Anti-CHOP	Monoclonal	2832	Cell Signaling[4]	1:500
Anti-eIF2α	Monoclonal	5324	Cell Signaling[4]	1:1000
Anti-GFP	Monoclonal	2956	Cell Signaling[4]	1:1000
Anti-IRE1α	Monoclonal	3294	Cell Signaling[4]	1:1000
Anti-PERK	Monoclonal	3192	Cell Signaling[4]	1:1000
Anti-XBP1s	Monoclonal	12782	Cell Signaling[4]	1:1000
Anti-Phospho-eIF2α	Monoclonal	3597	Cell Signaling[4]	1:1000
Anti-Phospho-PERK	Monoclonal	3179	Cell Signaling[4]	1:1000

[1] Abcam Plc, Cambridge, UK. [2] Santa Cruz Biotechnology, California, USA. [3] Sigma-Aldrich, St Louis, USA. [4]Cell Signaling Technology, Beverly, Massachusetts, USA.

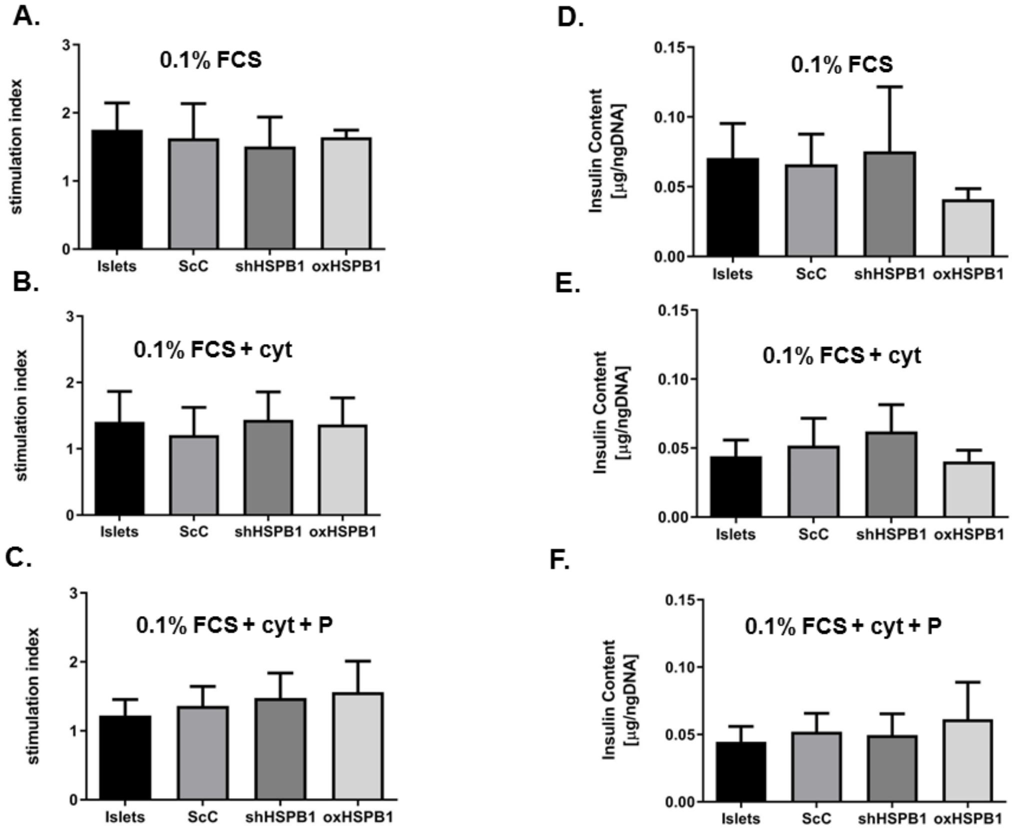

Supplementary Figure S1. Secretory function and insulin content of primary cultures of mouse islets. (A-C) Functional assay of mouse islets: mouse islets, HSPB1 silenced (shHSPB1) or not (ScC) or mouse islets overexpressing HSBP1 (oxHSPB1) were treated with (**A**) vehicle, (**B**) pro-inflammatory cytokines (cyt) in the absence or (**C**) in the presence of PRL (P) and incubated for two hours in the presence of media containing low or high glucose concentration. The secretory function of the cells was assayed and expressed as stimulation index. Stimulation index: (Concentration of insulin secreted in medium containing 16.7mM of glucose)/ (Concentration of insulin secreted in medium containing 2.8mM of glucose). (**D-F**) Insulin content in primary culture of mouse islets. Total insulin content of mouse islets, HSPB1 silenced (shHSPB1) or not (ScC) or mouse islets overexpressing HSBP1 (oxHSPB1) were treated with (**D**) vehicle, (**E**) pro-inflammatory cytokines (cyt) in the absence or (**F**) in the presence of PRL (P). The data were normalized by the total DNA content per sample. Results are presented as mean ± SEM (n= 3 independent experiments). The results displayed no significant differences between them. Islets: control mouse islets; ScC: islets infected with scramble shRNA, control C; shHSPB1: islets infected with short

hairpin for HSPB1 and oxHSPB1: islets transiently transfected with pEGFPhsp27 wt FL.

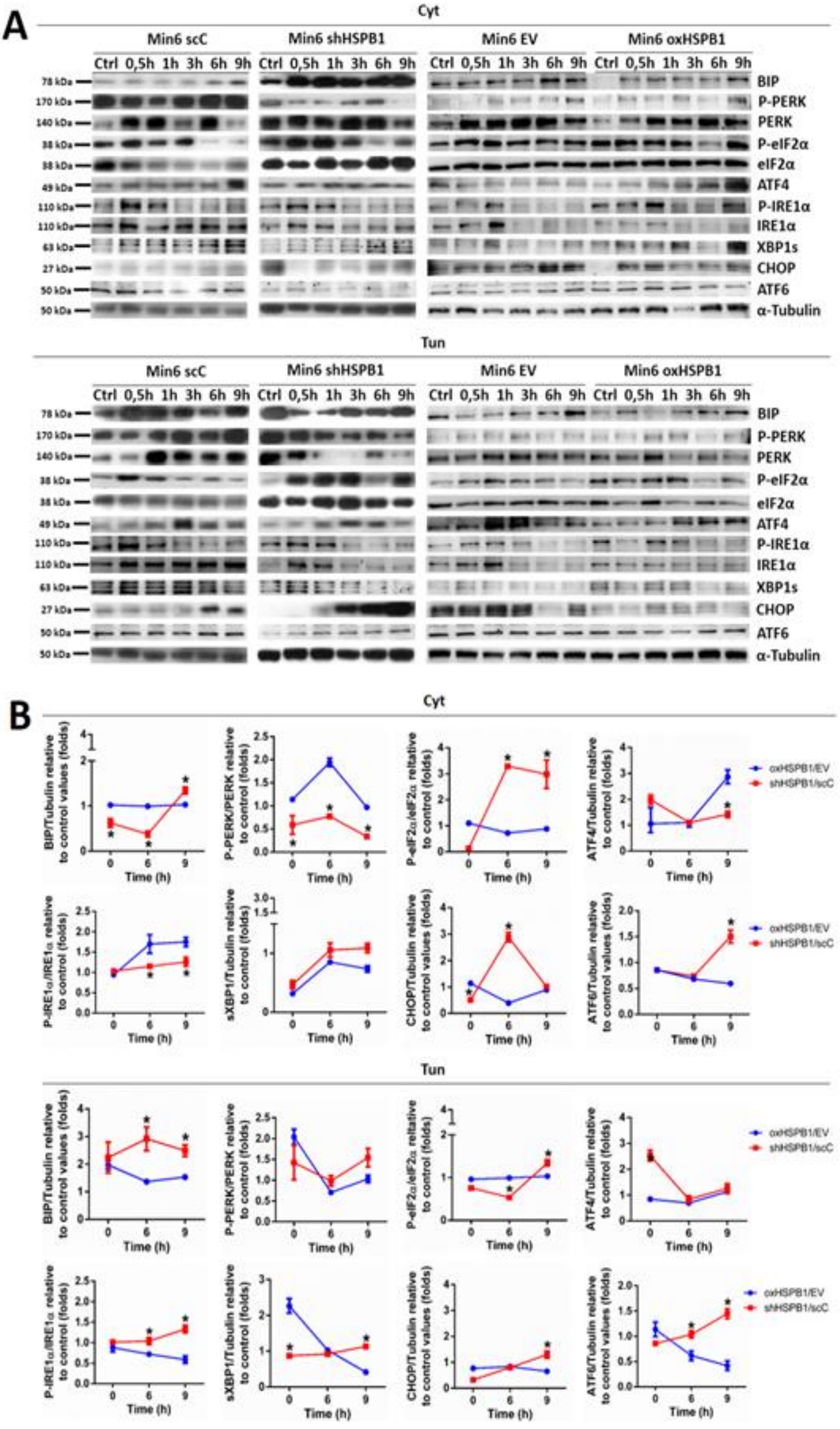
A
Cyt
Min6 scC
Min6 shHSPB1
Min6 EV
Min6 oxHSPB1
Ctrl 0,5h 1h 3h 6h 9h
78 kDa
170 kDa
140 kDa
38 kDa
38 kDa
49 kDa
110 kDa
110 kDa
63 kDa
27 kDa
50 kDa
50 kDa
BIP
P-PERK
PERK
P-eIF2α
eIF2α
ATF4
P-IRE1α
IRE1α
XBP1s
CHOP
ATF6
α-Tubulin
Tun
B
Cyt
BIP/Tubulin relative to control values (folds)
P-PERK/PERK relative to control (folds)
P-eIF2α/eIF2α relative to control (folds)
ATF4/Tubulin relative to control (folds)
P-IRE1α/IRE1α relative to control (folds)
sXBP1/Tubulin relative to control (folds)
CHOP/Tubulin relative to control values (folds)
ATF6/Tubulin relative to control values (folds)
Time (h)
oxHSPB1/EV
shHSPB1/scC
Tun

Supplementary Figure S2. Increased HSPB1 expression modulates the UPR in Min6 cells under ER stress induced by pro-inflammatory cytokines or tunicamycin. HSPB1 silenced (Min6 shHSPB1) or overexpressing Min6 cells (Min6 oxHSPB1) and their respective controls (Min6 scC, Min6 EV) were exposed after serum starvation, to a combination of cytokines (TNF-α 8 ng/mL, INF-γ 16 ng/mL, IL-1-β 1.6 ng/mL) or tunicamycin (15 μg/mL) for 0.5, 1, 3, 6 and 9 h. **(A)** Immunoblots of islets are shown as representative results. **(B)** Graphical representation of protein levels of ATF4, ATF6, BIP, CHOP, XBP1s and α-tubulin (used as a loading control), as well as phosphorylation state of PERK, eIF2α and IRE1α were analyzed by western blotting. After normalization of each protein to the corresponding α-tubulin, the data of the silenced HSPB1 cells (shHSPB1) or overexpressed (oxHSPB1) were plotted as the ratio between the values obtained in silenced or overexpressing cells and the one in their respective controls (scC or EV). *: $p < 0.05$ vs. oxHSPB1.

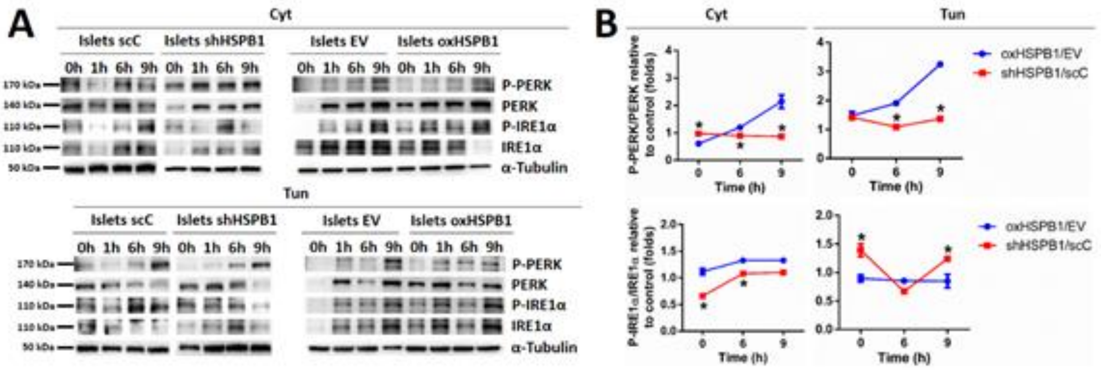

Supplementary Figure S3. Increased HSPB1 expression modulates PERK and IRE1α phosphorylation in mouse islets under ER stress induced by pro-inflammatory cytokines or tunicamycin. HSPB1 silenced (shHSPB1) or

overexpressing mouse islets (oxHSPB1) and their respective controls (scC, EV) were exposed after serum starvation, to a combination of cytokines (TNFα 8 ng/mL, INFγ 16 ng/mL, IL-1β 1.6ng/mL) or tunicamycin (15 μg/mL) for 0.5, 1, 3, 6 and 9 h. Phosphorylation levels of PERK and IRE1α were analyzed by western blotting. **(A)** Immunoblots are shown as representative results. Graphical representation presented as arbitrary densitometry units (AU). After the normalization of each protein to the corresonding α-tubulin, the data of the HSPB1 silenced (shHSPB1) or overexpressing cells (oxHSPB1) were normalized with their respective controls (scC or EV). Each data point represents means ± SEM from three replicates and at least three independent experiments were carried out for each cell type submitted to the different cell incubation conditions. *: p <0.05 vs. oxHSPB1/EV.

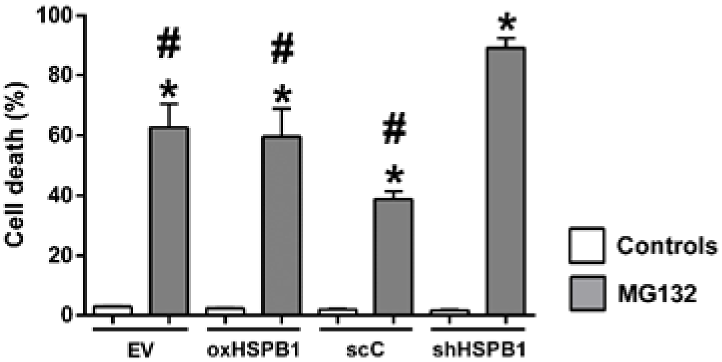

Supplementary Figure S4. The presence of HSPB1 promotes a higher cell death of murine pancreatic beta-cell with inhibited proteasome. Min6 cells overexpressing HSPB1 (Min6 oxHSPB1) and their respective controls (Min6 EV), were submitted to serum starvation (0.1% FCS) and were treated with the proteasome inhibitor MG132 (100 nM) for 16 h. Cell death was then evaluated

by microscopy. Results are presented as means ± SEM of three independent experiments. *: p<0.05 vs. control (vehicle), #: p <0.05 vs. shHSPB1.

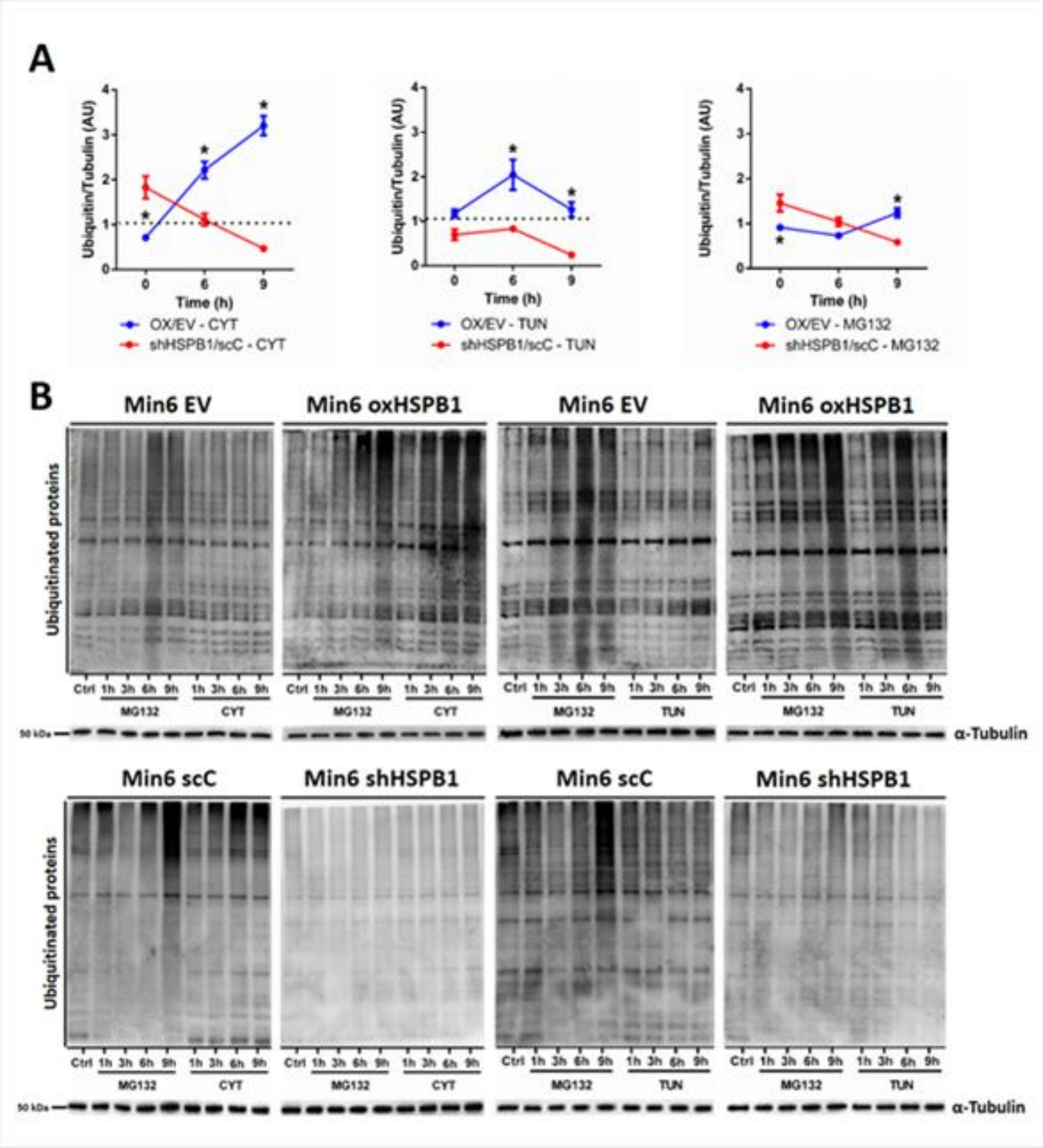

Supplementary Figure S5. HSPB1 levels modulates global protein ubiquitination in Min6 cells. HSPB1 silenced cells (shHSPB1) or overexpressing (oxHSPB1) and their respective controls (scC, EV) were serum starved and then exposed to combination of cytokines (TNF-α 8 ng/mL, INF-γ 16 ng/mL, IL-1-β 1.6 ng/mL), tunicamycin (15 μg/mL) or MG132 (2 μM) for 1, 3,

6 and 9 h. **(A)** Graphical representation of ubiquitinated proteins detection presented as arbitrary densitometry units (AU) after normalization to the corresponding α-tubulin. **(B)** Representative images of the immunoblots showing the levels of ubiquitinated proteins of Min6 cells treated with cytokines or tunicamycin. Each data point represents means ± SEM from three replicates and at least three independent experiments were carried out for each cell type submitted to the different cell incubation conditions; *: p<0.05 vs shHSPB1/scC.

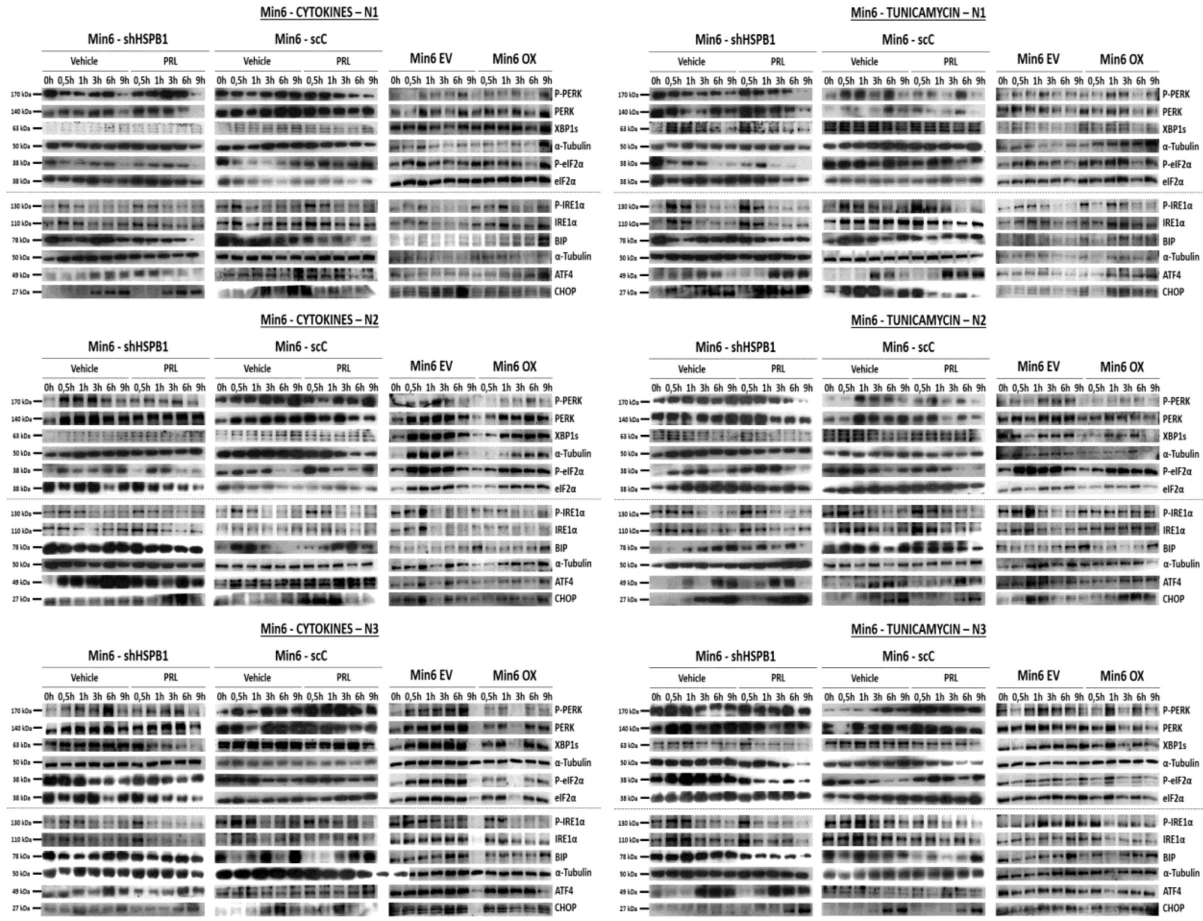

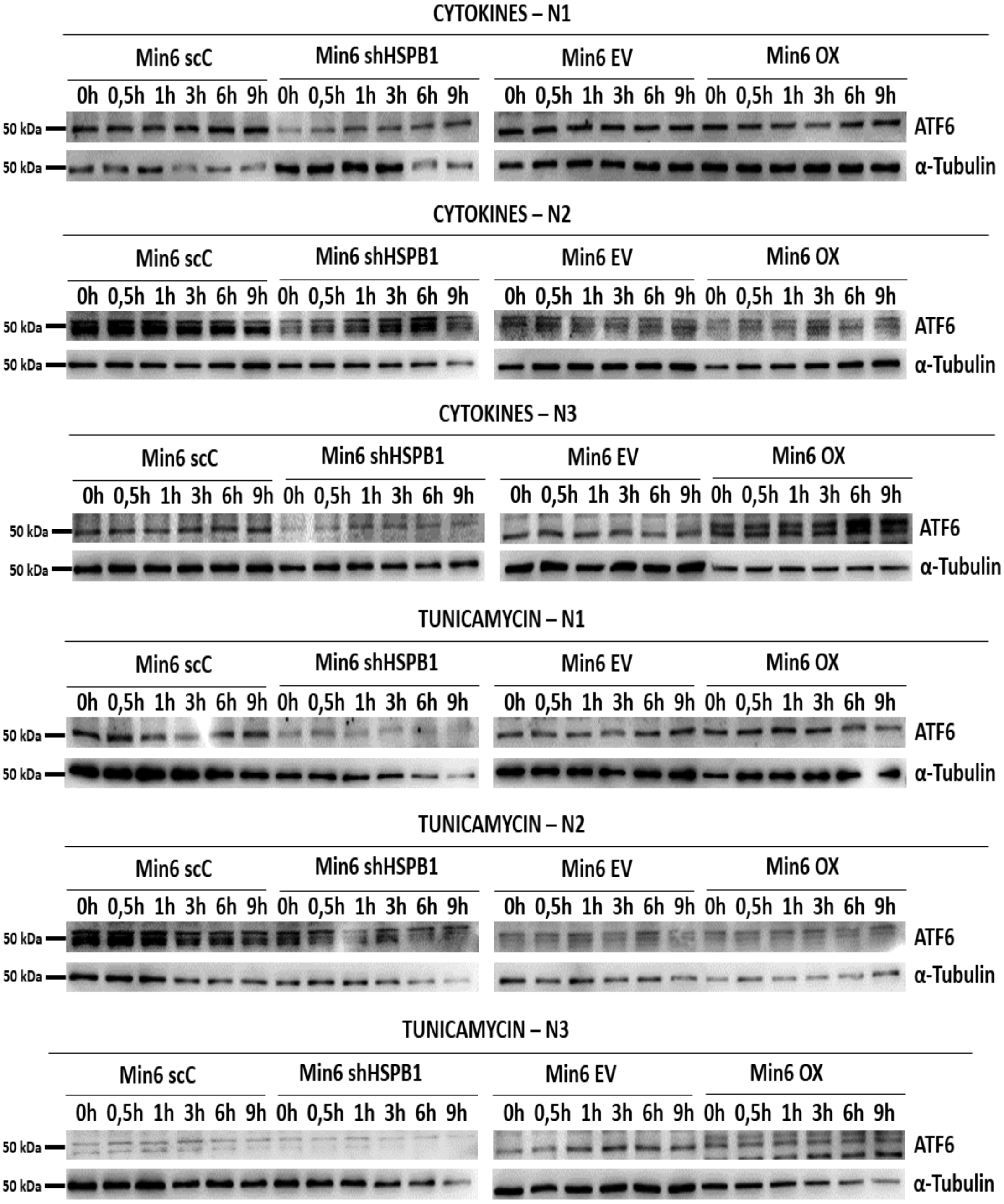
CYTOKINES – N1
Min6 scC
Min6 shHSPB1
Min6 EV
Min6 OX
0h 0,5h 1h 3h 6h 9h
50 kDa
ATF6
α-Tubulin
CYTOKINES – N2
CYTOKINES – N3
TUNICAMYCIN – N1
TUNICAMYCIN – N2
TUNICAMYCIN – N3

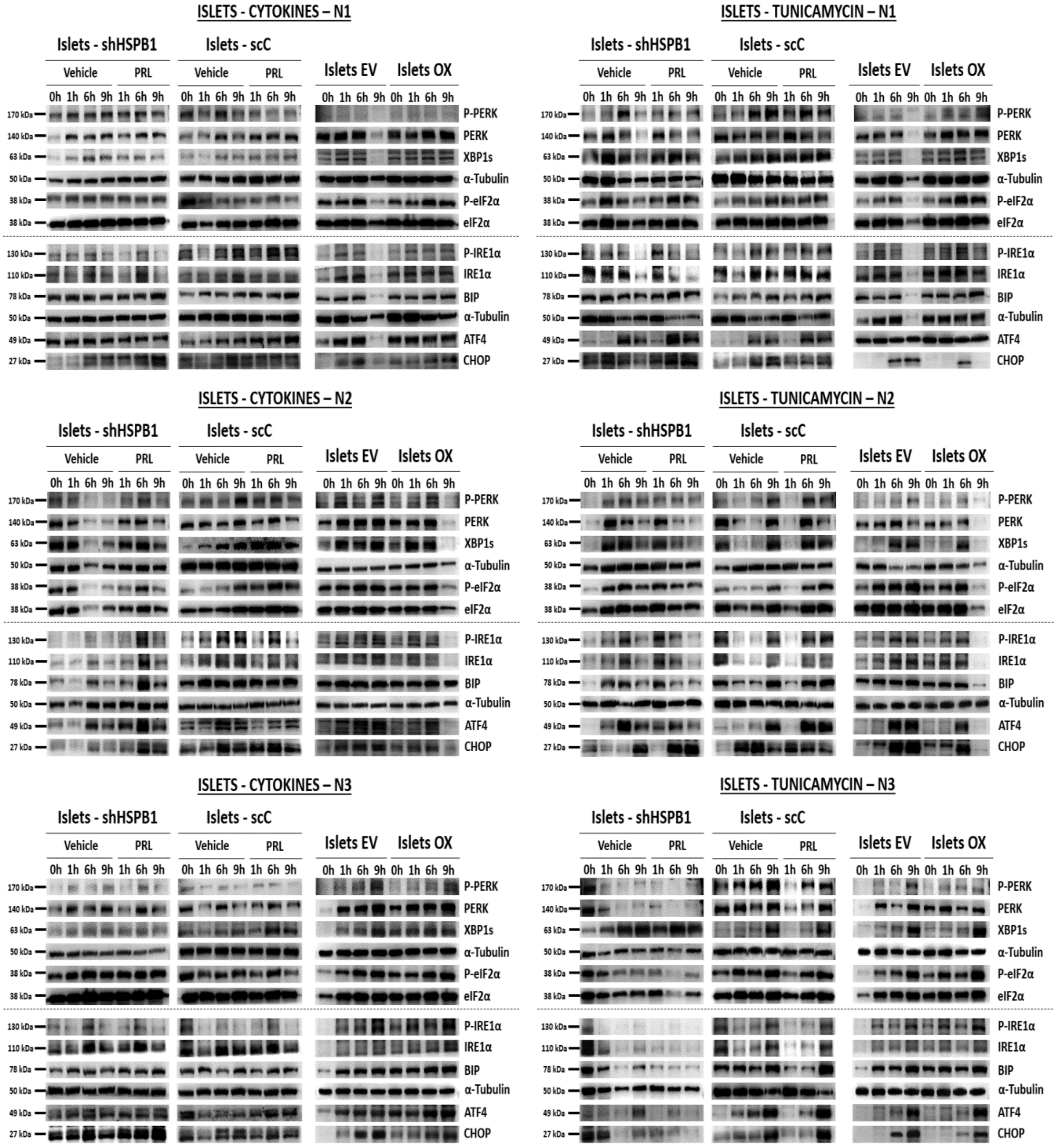
ISLETS - CYTOKINES – N1
ISLETS - TUNICAMYCIN – N1
ISLETS - CYTOKINES – N2
ISLETS - TUNICAMYCIN – N2
ISLETS - CYTOKINES – N3
ISLETS - TUNICAMYCIN – N3
Islets - shHSPB1
Islets - scC
Islets EV
Islets OX
Vehicle
PRL
0h 1h 6h 9h 1h 6h 9h
0h 1h 6h 9h 0h 1h 6h 9h
170 kDa
140 kDa
63 kDa
50 kDa
38 kDa
130 kDa
110 kDa
78 kDa
49 kDa
27 kDa
P-PERK
PERK
XBP1s
α-Tubulin
P-eIF2α
eIF2α
P-IRE1α
IRE1α
BIP
ATF4
CHOP

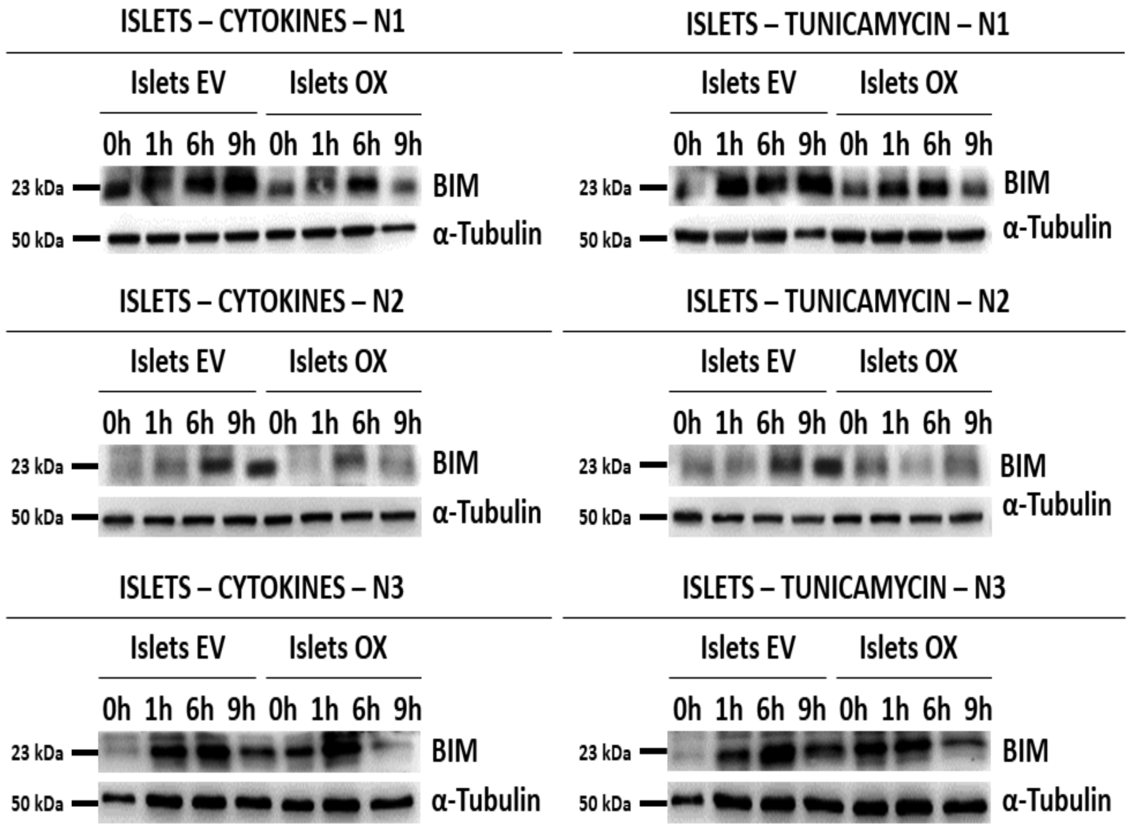

Supplementary Figure S6. Uncropped blots
